# Dual chromatographic analysis of pesticide residues in eggplants: a green approach to food safety monitoring

**DOI:** 10.1038/s41598-025-27091-w

**Published:** 2025-11-27

**Authors:** Amira M. Hegazy, Maha M. Abdelrahman, Mariam R. Kamel, Adel M. Ahmed

**Affiliations:** 1 Pharmaceutical Analytical Chemistry Department, Faculty of Pharmacy, Beni-Suef University, Alshaaheed Shehata Ahmad Hegazy St., Beni-Suef, 62514 Egypt; 2 Farshout Central Hospital, Qena, Egypt; 3 Department of Pharmaceutical Analytical Chemistry, Faculty of Pharmacy, South Valley University, Qena, 83523 Egypt

**Keywords:** HP-TLC, RP-HPLC, Chlorfenapyr, Abamectin, Fenpyroximate, Eggplant, Ecology, Plant sciences, Health care, Chemistry

## Abstract

The determination of pesticide residues is crucial for safeguarding human health, ensuring food safety, and contributing to environmental sustainability. Chlorfenapyr (CLF), abamectin (ABM), and fenpyroximate (FNP) are widely employed in eggplant (Solanum melongena L.) cultivation for pest control. This study presents the first reported simultaneous analysis of these pesticides using two validated chromatographic methods: high-performance thin-layer chromatography (HP-TLC, Method A) and reversed-phase high-performance liquid chromatography (RP-HPLC, Method B). Method A employed a mobile phase of methanol/chloroform/glacial acetic acid/triethylamine (7.00:2.50:0.50:0.10, v/v) with densitometric UV detection at 225 nm. The retention factors (Rf) for CLF, ABM, and FNP were 0.13, 0.34, and 0.85, respectively. Method B utilized a mobile phase of acetonitrile/5 mM ammonium acetate buffer (pH 4.0) (70:30, v/v) at a flow rate of 1.20 mL/min, with UV detection at 255 nm. The retention times for CLF, ABM, and FNP were 3.4, 6.1, and 8.4 min, respectively. Both methods were validated according to the key principles of ICH guideline Q2(R2) for the validation of analytical procedures, demonstrating robustness for assaying the ternary pesticide mixture in pure forms, commercial formulations (with excipients), and spiked eggplant samples. Both developed methods exhibited excellent detection and quantification capabilities. Method A demonstrated LODs ranging from 0.0006 to 0.0027 µg/band and LOQs from 0.0019 to 0.009 µg/band. Method B achieved even lower detection limits, with LODs from 0.0029 to 0.0296 µg/mL and corresponding LOQs from 0.0089 to 0.089 µg/mL for the target pesticides.

**Supplementary Information:**

The online version contains supplementary material available at 10.1038/s41598-025-27091-w.

## Introduction

### The imperative for pesticide residue monitoring

The environmental and health risks posed by pesticide residues have garnered significant global attention in both environmental sustainability and food safety research^1^. The potential for elevated pesticide levels remaining in food due to improper application has prompted governments and international organizations to implement strict maximum residue limits (MRLs) to protect consumer health.

### Eggplant as a key crop and its vulnerability

Eggplant (Solanum melongena L.) is a regionally and commercially significant crop and one of the most popular traditional foods in Egypt^2^, valued for its nutritional composition, including vitamins, proteins, and carbohydrates^3^. However, the susceptibility of eggplant fruits to insect pests often necessitates pesticide application by farmers to prevent substantial losses in productivity and quality^4^. Nevertheless, the extensive use of these pesticides can result in consumer exposure to potentially harmful residues^5,6^. Therefore, monitoring residues prior to human consumption is critical, necessitating the development of efficient, cost-effective, and simultaneous analytical methods to determine residues of commonly used agrochemicals like chlorfenapyr (CLF), abamectin (ABM), and fenpyroximate (FNP).

### Profile of the target pesticides

**Fig. 1:**
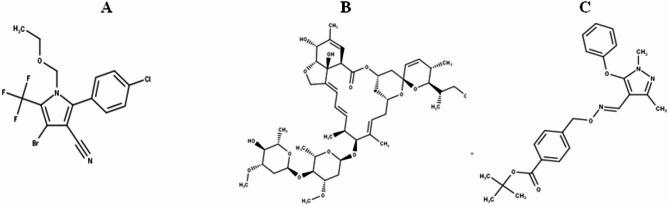
Chemical structures of **A**: CLF, **B**: ABM, and **C**: FNP.

1. CLF is Chemically named [4-bromo-2-(4-chlorophenyl)−1-ethoxymethyl-5-(trifluoromethyl)pyrrole-3-carbonitrile] (Fig. 1). It is a broad-spectrum pyrrole insecticide-acaricide that presents as a white-to-tan powder. It functions as a pro-insecticide, undergoing metabolic activation into a toxic compound upon pest ingestion, while demonstrating a relatively low impact on beneficial predatory insects^7^. It provides effective control against various pest species, including Liriomyza sp., Frankliniella occidentalis, Spodoptera exigua, and Spodoptera litura^8^.
2. ABM is a macrocyclic lactone (Fig. 1), composed of a mixture of avermectin B1a (80%) and B1b (20%) homologs^9,10^. It functions as a broad-spectrum, systemic insecticide that effectively controls mites and various mobile insect stages on fruits and vegetables^11^. Since its introduction in the 1980 s, the use of ABM has significantly increased. Its primary variations, B1a and B1b, differ in their methylation patterns^12^.
3. FNP is an acaricide characterized by an oxime-bearing pyrazole structure^13,14^ (Fig. 1). It exhibits high efficacy against larval stages by inhibiting mitochondrial electron transport^15^. Chemically designated as α-(4-phenoxyphenyl)-α, α-dimethyl-1 H-pyrazole-3-propanenitrile, FNP is a pyrazole acaricide and insecticide classified within the sulfonanilide group^16,17^.

### Analytical gap and literature review

A review of existing literature reveals numerous chromatographic methods for the determination of CLF, ABM, and FNP individually or in combination with other pesticide classes. For instance, CLF has been determined via HPLC in various vegetables like cabbage^18–20^ and green beans^8^, in formulations^21^, and in water by GC^22^. ABM has been extensively estimated, including recent HPLC methods in 2024 for lake water, milk, and peach juice^23^, and previously in avocados^24,25^, citrus fruits^26,27^, and other matrices^28–32^. Similarly, FNP has been determined by HPLC with other pesticides^33,34^ and in various samples including apples^35,36^, grapes^37^, honey^38^ and surface water^39^.

### Rationale and novelty of the present study

Critically, no method has been reported for the simultaneous quantification of this specific ternary combination (CLF, ABM, and FNP). This represents a significant analytical gap, as these pesticides are commonly used in combination or rotation in eggplant cultivation to combat complex pest infestations and prevent resistance. Consequently, current monitoring programs require laboratories to run multiple, separate single-analyte methods, which are time-consuming, resource-intensive, and costly. As pesticides are among the most toxic contaminants in fruits and vegetables^40^, and with a growing trend towards multi-residue analysis^41–44^, there is a clear need for a simultaneous method. This gap motivated our research team to develop two selective and sensitive chromatographic methods: high-performance thin-layer chromatography (HP-TLC, Method A) and reversed-phase high-performance liquid chromatography (RP-HPLC, Method B) for the estimation of this target mixture.

### Strategic application of dual chromatographic methods

The strategic application of both techniques addresses different needs in the analytical workflow. HP-TLC serves as an efficient and economical preliminary screening tool with high throughput and minimal solvent consumption, making it ideal for initial monitoring. Conversely, RP-HPLC is preferred for its superior sensitivity, automation, and regulatory compliance for precise confirmatory quantification. This dual approach allows laboratories to maximize efficiency and resource utilization, using HP-TLC for initial screening followed by RP-HPLC confirmation for non-compliant samples, thereby balancing affordability with analytical rigor. This is particularly relevant in the current regulatory climate, where MRLs for such substances are under continuous review^45^. The newly developed methods were successfully applied to determine the investigated pesticides simultaneously in pure forms, commercial formulations, and spiked eggplant samples, fulfilling a critical need for monitoring this specific pesticide combination.

## Experimental

### Instruments

#### Method A

Data were acquired using a Camag TLC system that included a Linomat V auto-sampler and a TLC scanner III, operated with winCATS version 1.4.4.6337 software (CAMAG, Muttenz, Switzerland). The system requirements included a deuterium lamp as the radiation source, absorbance mode for scanning, output of the chromatogram as an integrated peak area, slit dimensions of 4 × 0.45 mm, a Linomat V autosampler with a 100 µL syringe for sample application, and a scanning speed of 20 mm/s.

Pre-coated 0.25-mm silica gel 60 F254 TLC aluminum plates (20 × 20 cm; Merck, Germany) were used as the stationary phase. A 4000-rpm electrical centrifuge (Zjmzym, China), a 0.1–100 µL Rongtai variable-volume micropipette (Shanghai, China), and a 250 VM vortex mixer from Hwashin (Seoul, Korea) were used.

#### Method B

An Agilent Technologies 1260 Infinity series LC system connected to a 1260 UV-VIS detector was used, equipped with an Agilent Technologies Eclipse Plus C18 column (25 cm x 4.6 mm i.d., 5 μm particle size).

#### Sample preparation

Other equipment included for sample preparation.

- Rotavapor R-300 (BUCHI Co., Switzerland),
- Digital Ultrasonic Cleaner (USA),
- Benchtop pH meter BP-3001 (TTBH Pte Ltd, Singapore),
- Vortex mixer VSM-3 (Shelton Scientific, USA),
- Thermo Scientific Megafuge-8 Benchtop Centrifuge (UK),
- djustable micropipette with variable volumes (0.10–1000 µL, Shandong, China), &.
- Vertical ultrafreezer ULF500 (−86 °C, Infrico medicare, Spain).

### Material and reagents

#### Pure samples

The concerned pesticides (CP) were purchased from Sigma-Aldrich (Talat Harb, Cairo, Egypt). Their purities were certified to be 99.21%, 99.05%, and 99.10% for CLF, ABM, and FNP, respectively, according to the supplier’s certificate of analysis. (Note: The text said “99.21, 99.05, 99.10 and 99.47%” which lists four values for three pesticides. I corrected this to three values. Please verify the correct purities.)

#### Commercial formulations

- Baylora^®^ (Batch No. 502023), labeled to contain 24% w/w CLF, was manufactured by Hebei Guanwang Agro Chemical, China.
- Aquachem^®^ (Batch No. 2023115), labeled to contain 5.7% w/w ABM, was manufactured by Jialingsu Subin Agro Chemical, China.
- Ortus Super^®^ (Batch No. 2051947), labeled to contain 5% w/w FNP, was manufactured by Shoura Chemicals, Cairo-Alexandria Desert Road, Egypt.

#### Chemicals and reagents

##### Method A

Methanol (MeOH) and acetonitrile (ACN) were HPLC-grade reagents purchased from Sigma-Aldrich Chemie GmbH, Germany.

##### Method B

Acetone (ACT), chloroform (CFM), glacial acetic acid (GAA), ammonium acetate (AA), and triethylamine (TEA) were of analytical grade (El-Nasr Pharmaceutical Chemicals Co., Cairo, Egypt). De-ionized water (DW) was obtained from SEDICO Co., Egypt.

### Standard solutions and sample preparations

#### Standard solutions

Stock standard solutions of CLF, ABM, and FNP were prepared in MeOH at a concentration of 1000 µg/mL. Working standard solutions (100 µg/mL) were prepared by appropriate dilution. All stock standard solutions were freshly prepared on the day of analysis and stored in a refrigerator for no longer than 24 h.

#### Field sampling and pesticide application

Field Trial Design: A controlled field trial was conducted in a representative eggplant (*Solanum melongena* L.) cultivation area in the El-Fayoum governorate, Egypt, during the winter season of 2024–2025. The trial plot area was 0.5 hectares.

Pesticide Application Regime: The commercial formulations Baylora^®^ (CLF), Aquachem^®^ (ABM), and Ortus Super^®^ (FNP) were applied as a tank mixture at their recommended field rates (250 g/L for CLF, 50 mL/L for ABM, 100 mL/L for FNP) using a motorized knapsack sprayer. Application was performed at the fruiting stage according to standard agricultural practices. Environmental conditions during application were recorded: temperature was, 25 ± 2 °C, relative humidity was 60 ± 5%, and wind speed was negligible.

Sample Collection: Sampling was performed according to the guidelines of the Joint FAO/WHO Meeting on Pesticide Residues (JMPR)^46^. Samples were collected at six intervals: 2 h (0-day), and 1, 3, 7, 10, and 14 days after application (DAA). This allows for the construction of dissipation curves.

At each interval, three replicate samples were collected from randomly selected plants across the treated plot. Each replicate sample consisted of approximately 1 kg of fresh, ripe, marketable eggplants, yielding a total of ~ 3 kg per time point. To avoid cross-contamination, samples were handled with clean gloves. A control sample was collected from an untreated plot maintained under the same agronomic conditions before the application of pesticides.

Immediately after collection, samples were labeled, placed in sterile polyethylene bags, transported to the laboratory in an icebox (maintained at ~ 4 °C), and processed upon arrival.

#### Spiked eggplant (quality control) sample

To confirm the functional applicability of the calculated Limits of Quantification (LOQ) within the complex matrix, a single quality control (QC) sample was prepared by spiking the blank eggplant matrix extract with the three analytes at a concentration equal to the highest LOQ value determined by both methods.

#### Commercial formulations

An amount equivalent to 100 mg of CLF, ABM, and FNP from Baylora^®^ powder, Aquachem^®^, and Ortus Super^®^, respectively, was accurately weighed and transferred separately to 100-mL volumetric flasks. Then, 75 mL of MeOH was added to each flask. The solutions were sonicated for 20 min, cooled, diluted to volume with MeOH to obtain 1 mg/mL stock solutions, and then filtered. Appropriate dilutions of the stock solutions were made to obtain 100 µg/mL working solutions.

### Procedures

#### Chromatographic conditions

##### Method A

HP-TLC separation was performed on (20 × 10 cm) silica gel 60 F254 pre-coated plates (0.25 mm thickness). The plates were pre-washed with MeOH and activated at 100 °C for 15 min. Samples were applied as 6-mm bands, spaced 5 mm apart and 10 mm from the plate’s lower edge, using a CAMAG Linomat-V automated sampler.

Ascending linear development was performed in a chromatographic tank pre-saturated for 30 min with the mobile phase MeOH: CFM: ACT: GAA: TEA (7.00:2.50:0.50:0.10:0.10, v/v). Development proceeded to a distance of 9 cm at ambient temperature. After air drying, the plates were scanned using a CAMAG HP-TLC scanner at 255 nm. Data acquisition and analysis were performed using WINCATS software with a deuterium lamp. The peak areas were recorded.

##### Method B

Separation was performed using an Agilent Zorbax Eclipse Plus-C18 column (250 × 4.6 mm, 5 μm). The mobile phase consisted of ACN:5 mM AA buffer (70:30, v/v), with the buffer pH adjusted to 4.00. The flow rate was 1.20 mL/min, and UV detection was performed at 255 nm.

Preparation of 5 mM AA buffer (1 L): 385.40 mg of AA (CH₃COONH₄, purity > 99%) was weighed into a beaker, dissolved in approximately 800 mL of DW, equilibrated at room temperature (20–25 °C), and adjusted to pH 4.0 with acetic acid before diluting to 1 L with DW.

#### Construction of calibration curves

##### Method A

Aliquots from standard stock solutions (1 mg/mL) of CLF (0.005–0.5 µg), ABM (0.002–0.1 µg), and FNP (0.01–0.12 µg) were accurately transferred to a set of 10-mL volumetric flasks and diluted to volume with MeOH. A 10 µL volume of each prepared solution was applied to HP-TLC plates (20 × 10 cm, pre-washed with MeOH and dried at 60 °C for 5 min) as 6-mm bands using a Camag Linomat IV applicator. The bands were applied 10 mm from the bottom edge and 5 mm apart.

Linear ascending development was performed in a chamber pre-saturated for 30 min with MeOH: CFM: ACT: GAA: TEA (7.00:2.50:0.50:0.10:0.10, v/v) as the mobile phase. UV detection was performed at 255 nm.

##### Method B

Accurate aliquots of CLF, ABM, and FNP working standard solutions (100 µg/mL) were transferred into separate sets of volumetric flasks to prepare solutions with concentration ranges of 0.02–2.00.02.00, 0.009–1.00.009.00, and 0.09–1.20 µg/mL, respectively. Triplicate injections were performed for each concentration. The calibration curve was constructed by plotting the integrated peak area against the corresponding concentration, and regression equations were derived.

Chromatographic separation was performed in isocratic mode on a C18 column with a mobile phase of ACN: 5 mM AA buffer (70:30, v/v, pH 4.00). The flow rate was 1.20 mL/min, and UV detection was at 255 nm.

#### Repeatability

A minimum of nine determinations covering the specified procedure range (three concentrations/three replicates each) was investigated.

#### Reproducibility

Reproducibility was assessed through an inter-laboratory trial. Investigation of reproducibility is not typically required for regulatory submission but should be considered for the standardization of an analytical procedure, such as for inclusion in pharmacopoeias.

#### Field sample preparation

The Eggplant peel extracts were prepared from samples collected in Egypt in Feb. 2025. Peels were manually removed, chopped, and a representative 15.0 g ± 0.1 g portion of the homogenate was weighed into a 50-mL Teflon centrifuge tube then combined with 25 mL of CAN and centrifuged for 4 min. After adding 5 g of anhydrous sodium sulfate, the mixture was centrifuged at 6000 rpm for 14 min at 18 °C. The resulting supernatant was collected and concentrated to 3 mL under vacuum at 40 °C using a rotary evaporator. The concentrated extract was then filtered through a 0.45 μm filter. Stringent laboratory practices, including the use of clean equipment, were employed throughout the preparation process to minimize cross-contamination. Samples were analyzed immediately after preparation.

#### Application to commercial formulations

The procedure described under the construction of the calibration curve was followed for the prepared solutions of the commercial formulations. The concentrations of the CP were calculated from the corresponding regression equations.

#### Application to field samples

The procedure described under the construction of the calibration curve was followed for the prepared eggplant samples. The concentrations of the CP were calculated from the corresponding regression equations.

To prevent cross-contamination, all equipment was meticulously cleaned between samples, and the analysis order included solvent blanks and control matrix samples to monitor for any carry-over, which was not observed.

## Results and discussion

### Method development and optimization

#### Method A

**Fig. 2:**
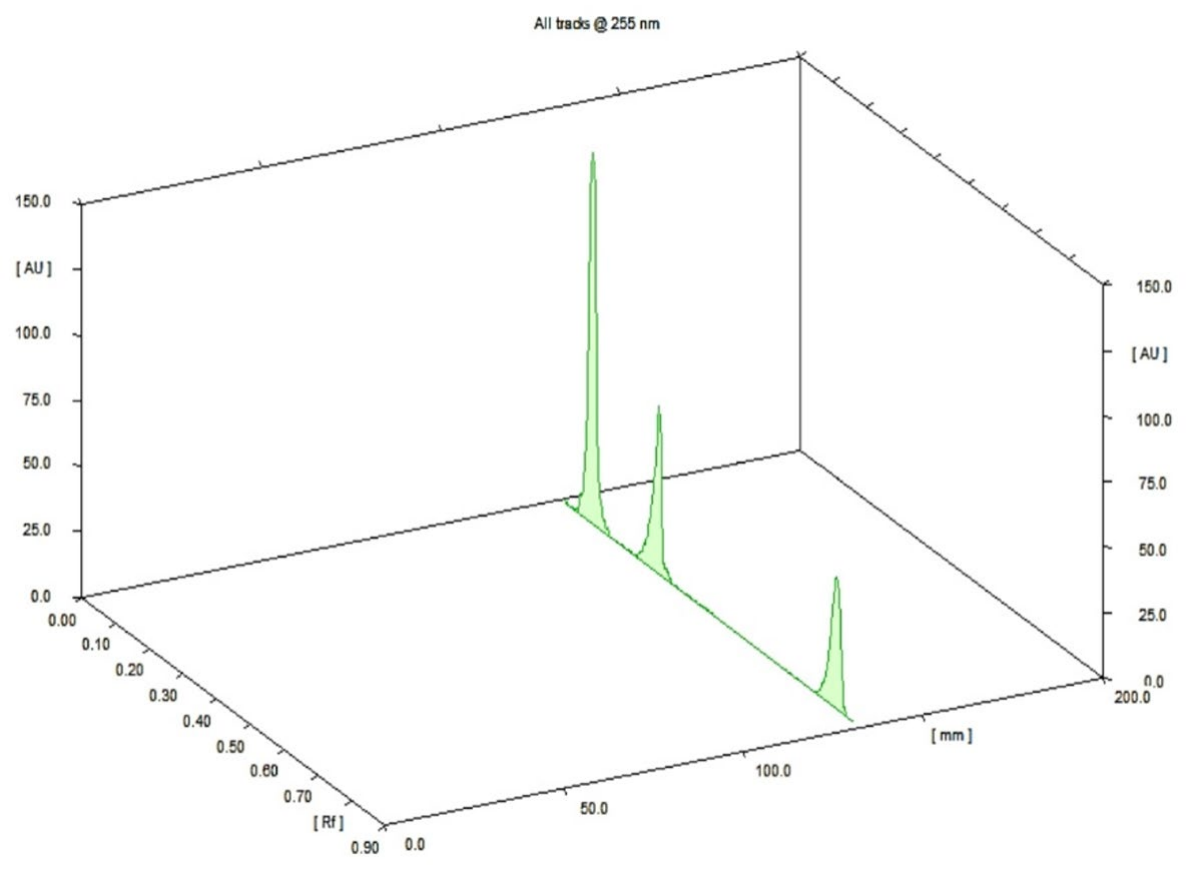
3D HP-TLC-densitogram of the mixture: CLF (R_f_=0.13), ABM (R_f_=0.34), and FNP (R_f_=0.85) using MOL: CM: AN: GAA: TEN (7.00: 2.50: 0.50: 0.1, v/v) as a MP and UV detection at 255 nm.

Optimization of the chromatographic separation for the ternary pesticide mixture involved evaluating several mobile phase compositions. Initial trials with MeOH: CFM (1:9 and 4:6, v/v) and hexane: MeOH (8:2 and 4:6, v/v) yielded unsatisfactory resolution. A subsequent attempt using MeOH: CFM: acetic acid (7.00:2.50:0.20, v/v/v) resulted in moderate separation but exhibited peak tailing. Optimal separation was achieved with a mobile phase consisting of MeOH: CFM: ACT: GAA: TEA (7.00:2.50:0.50:0.10:0.10, v/v). Scanning at 255 nm was employed to maximize sensitivity. The chromatographic chamber was pre-saturated with the mobile phase for 30 min to ensure homogeneity and minimize volatility. Under these conditions, the Rf values for CLF, ABM, and FNP were determined to be 0.13, 0.34, and 0.85, respectively (Fig. 2).

#### Method B

Several mobile phases were tested using an Agilent Zorbax Eclipse Plus-C18 (250 × 4.6 mm, 5 μm) column. Starting with MeOH: DW (90:10, v/v), overlapping broad peaks for CLF and ABM were obtained. Adjusting the ratio of MeOH to DW from (80:20, v/v) to (70:30, v/v) and finally (60:40, v/v) did not resolve the overlapping CLF and ABM peaks. Using MeOH:0.02 M Na₂HPO₄ buffer (70:30, v/v, pH 4 with ortho-phosphoric acid) also failed to resolve the peaks and introduced significant noise in the chromatogram between 2.50 and 5.25 min. Replacing the phosphate buffer with 5 mM AA buffer improved the resolution but some noise remained. Replacing MeOH with ACN minimized the noise but further improvement was needed. Another system, ACN:5 mM AA buffer (90:10, v/v, pH 5.60), resulted in good separation but FNP eluted at 14.00 min and the ABM peak showed tailing. Finally, ACN:5 mM AA buffer (70:30, v/v, pH 4.00) provided good resolution and symmetric peaks for all analytes, with retention times of 3.4, 6.1, and 8.4 min for CLF, ABM, and FNP, respectively.

**Fig. 3:**
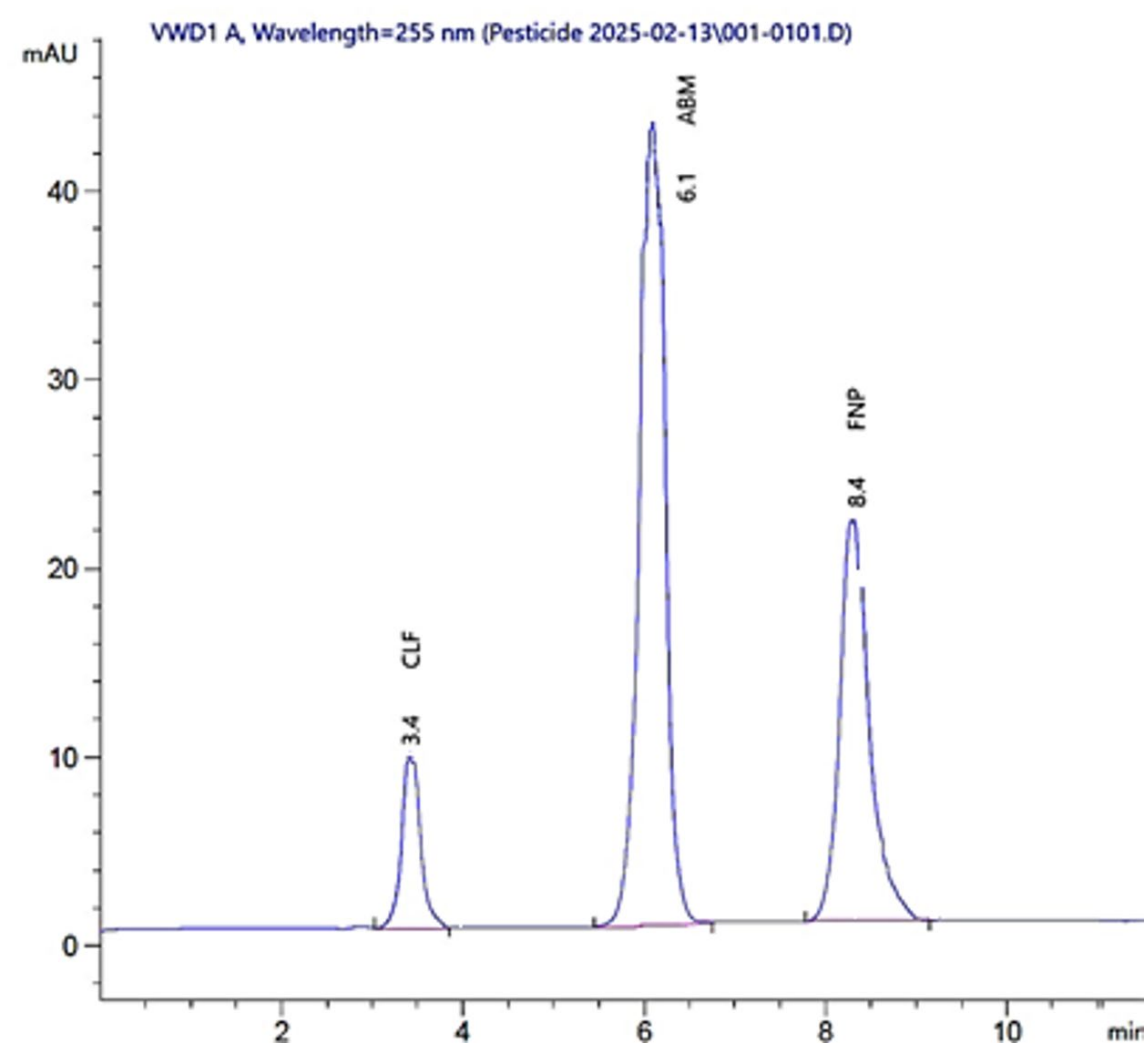
RP-HPLC chromatogram of the mixture: 0.40 µg/mL CLF, 1.00 µg/mL ABM, and 1.20 µg/mL FNP using AN: 5 mM AA buffer (70: 30, v/v), pH adjusted to 4.00, Flow rate 1.20 mL/min, and UV detection at 255 nm.

The flow rate was adjusted from 0.50 to 2.00 mL/min; complete separation within an acceptable time was achieved at 1.20 mL/min. Scanning was performed at various wavelengths (215, 220, 225, 230, 240, 255, 260, and 270 nm). A wavelength of 255 nm provided the best sensitivity for the CP with the least noise, as depicted in Fig. 3. The run time was short, at less than 10 min.

### Method validation

Both methods were validated according to the key principles of ICH guideline Q2(R2)^47^ for the validation of analytical procedures. The validated parameters included linearity, accuracy, precision (repeatability and intermediate precision), specificity, LOD, LOQ, and robustness. It should be noted that reproducibility, as defined by an inter-laboratory trial, was not conducted as the primary focus of this work was on method development and initial validation.

#### Linearity

The linearity of an analytical method is crucial for ensuring the detector response is directly proportional to the analyte concentration across a defined range, allowing for accurate quantification. For reliable pesticide residue analysis, the linear range must cover all expected concentrations, from the LOQ to the highest anticipated residue levels. Crucially, the established MRLs for specific pesticides, such as those for CLF (0.821 mg/kg), ABM (0.05 mg/kg), and FNP (0.3 mg/kg), are officially declared and regularly updated by regulatory bodies like the Codex Alimentarius Commission and the European Union^48–50^. Therefore, it is imperative that the developed analytical method demonstrates excellent linearity across a range that encompasses these MRLs, as shown in Table 1.

#### Accuracy

Accuracy was assessed by calculating the percent recovery (%R) of known amounts of pure CP using the corresponding regression equations. Acceptable recovery percentages were obtained for both methods (Table 1). The standard addition technique was performed by adding known amounts of pure CP to their pharmaceutical formulations; good recoveries were obtained, showing no interference from excipients (Table 2).

#### Precision

##### Repeatability

Three different concentrations of pure pesticides were analyzed in triplicate (intra-day) using Methods A and B. Good RSD% values were obtained, verifying the methods’ repeatability (Table 1).

##### Intermediate precision

The procedure was repeated inter-day (three consecutive days) for the analysis of the three chosen concentrations. Acceptable RSD% values were obtained (Table 1).

#### Specificity

Specificity of the methods was critically assessed by comparing chromatograms of:

- Standard solutions of the individual pesticides and the ternary mixture.
- Blank eggplant extract (control sample) to identify any potential co-eluting matrix interferences.
- Eggplant extract spiked with the target pesticides at the LOQ and MRL levels.

As shown in Fig. 2 (method A) and Fig. 3 (method B), the blank matrix samples showed no significant interfering peaks at the retardation factors (Rf = 0.13, 0.34, and 0.85) or retention times (Rt = 3.4, 6.1, and 8.4) corresponding to CLF, ABM, or FNP.

Furthermore, the peaks for all three analytes in the spiked samples were well-resolved and symmetrical, confirming the methods’ selectivity in the presence of the complex eggplant matrix. The high percentage recovery values (Table 1) further corroborate the lack of matrix-induced enhancement or suppression.

#### Limits of detection and quantitation (LOD and LOQ)

For Method A, LOD and LOQ were based on the standard deviation of the response and the slope of the calibration curves: LOD = 3.3 × SD/slope; LOQ = 10 × SD/slope. LOD and LOQ were also calculated using a visual non-instrumental method according to ICH recommendations^47^. For Method B, the low LOD and LOQ values demonstrate the high sensitivity of the proposed method (Table 1).

#### Robustness

The robustness of the proposed methodologies was verified by introducing small, deliberate changes in the chromatographic conditions (e.g., change in acetic acid amount ± 0.02%, saturation time ± 5 min in Method A; change in organic modifier ± 1%, flow rate ± 0.05 mL/min in Method B). These changes did not significantly affect the Rf values, peak symmetry, or peak area (Table 1).

#### System suitability

Overall system suitability testing was performed. Acceptable results for the system suitability parameters are shown in Table 1.

### Application to commercial formulations

Methods A and B were applied to determine the CP in their commercial formulations. The acceptable recovery values verified the efficacy of the proposed methods for determining the pesticides in their commercial formulations (Table 2).

### Field sample analysis

Field sample analysis showed initial concentrations of the studied pesticides (CLF, ABM, and FNP) to be 2.09, 0.06, and 0.08 mg Kg⁻¹ on the first day, which decreased to 0.082, 0.003, and 0.0108 mg Kg⁻¹ by day 10, respectively. These values are within the recommended doses for human health and vegetable productivity (Table S1).

Analysis of the blank control samples collected from the untreated plot confirmed the absence of any detectable residues of the target pesticides (concentrations below the LOD for all analytes), ensuring that the results from the treated plot were due solely to the applied pesticides and not background contamination.

### Validation of LOQ in eggplant matrix

The calculated Limits of Quantification (LOQs) for all three analytes were functionally confirmed in the eggplant matrix to establish the lowest concentration that could be accurately and precisely quantified. A dedicated LOQ confirmation samples were prepared by spiking the blank eggplant matrix extract at a concentration equal to the highest calculated LOQ value among the three analytes. These spiked samples were analyzed in three replicates (*n* = 3) alongside the other validation samples, by the developed methods. Table^3^ presents the mean recoveries, standard deviations (SD), and relative standard deviations (RSD) for the quality control (QC) concentration with three replicate. This detailed data clearly validates the method’s excellent reproducibility (all RSDs < 2.5%) and accuracy (recovery 95–105%) within the complex eggplant matrix, directly supporting the feasibility of the claimed LOQs. This result, which easily meets the validation criteria for trace analysis, unequivocally demonstrates that the new methods are both accurate and reproducible at the quantification limit within the realistic matrix environment.

### Statistical analysis

The results obtained from the proposed methods for the assay of pure samples of CLF, ABM, and FNP were statistically compared with those obtained from reference HPLC methods^7,28^, and^35^.

A pair of hypothesis tests was employed for each compound to compare the accuracy and precision of the proposed methods against the reference method:

Student’s t-test was used to compare the means (a test for bias/accuracy). A calculated t-value below the theoretical critical value (at *p* = 0.05) indicates that there is no statistically significant difference between the mean results obtained by the two methods.

F-test was used to compare the variances (a test for precision). A calculated F-value below the theoretical critical value (at *p* = 0.05) indicates that there is no statistically significant difference in the precision of the two methods.

The calculated t and F values for all three analytes, as shown in Table 3, were found to be below the theoretical critical values. This provides statistical evidence that the proposed methods exhibit equivalent accuracy and precision to the established reference method, with no significant systematic error or difference in reproducibility. This statistical comparison is crucial as it validates the reliability of the new simultaneous method against a proven benchmark.

### Greenness considerations

#### A comparative evaluation of HP-TLC and RP-HPLC in terms of solvent consumption and waste

When considering the environmental impact of chromatographic methods for pesticide residue analysis, both (HP-TLC) and (RP-HPLC) present distinct advantages and disadvantages in terms of their “greenness.” While RP-HPLC offers high resolution and sensitivity, its significant environmental drawback is its high consumption of organic solvents like acetonitrile and methanol, which generates large volumes of hazardous waste^51^. This necessitates efforts to “green” RP-HPLC by reducing column dimensions, flow rates, and run times, or by exploring less toxic “green solvents,” though these can have specific limitations.

In contrast, HP-TLC generally offers a more environmentally friendly profile. It inherently uses significantly less mobile phase solvent than HPLC, as the solvent moves by capillary action on a thin layer plate, directly translating to reduced solvent consumption and waste generation. Furthermore, HP-TLC’s ability to run multiple samples simultaneously on a single plate contributes to higher throughput and potentially lower energy consumption per sample. Therefore, for pesticide residue analysis, while RP-HPLC remains critical for its precision and scope, strategies like miniaturization and the adoption of HP-TLC-based approaches are increasingly valuable for mitigating the environmental footprint associated with routine pesticide residue analysis.

#### Sustainability assessment of the proposed methods and comparison with reported HPLC methods

To provide a quantitative assessment of these principles, the greenness of both proposed methods was evaluated using the Analytical GREEnness (AGREE) metric^52^. The AGREE calculator provides a comprehensive score based on the 12 principles of GREEN analytical chemistry, ranging from 0 (not green) to 1 (fully green).

The sustainability of the proposed analytical methods was comprehensively evaluated and benchmarked against existing HPLC methods by assessing their environmental impact and analytical performance. This evaluation was guided by green, red and blue chemistry principles, focusing on eco-friendliness, water conservation, and safety. Three modern assessment tools RAPI, BAGI, and AGREE were utilized to quantify and compare the methods’ sustainability and analytical quality by Prajapati et al.^53,54^.

##### Blueness profile evaluation

The proposed methods^7,28^, and^35^ showed higher blueness scores (80 and 82.5) and more dark-blue segments in the BAGI pictograms, indicating better ecological alignment compared to the reported HPLC methods, which scored lower (57.5 to 67.5) with more light-blue segments (Table S2).

##### Greenness profile evaluation

The proposed methods obtained AGREE scores of 72 and 77, indicating superior environmental sustainability compared to the reported methods, which yielded lower scores of 58, 52, and 55 (Table S2).

##### Red analytical performance index

Analytical quality and method robustness were additionally evaluated via the RAPI metric. The proposed methods achieved a high RAPI score of 80, signifying enhanced reliability and performance, surpassing the scores of previously reported methods (65, 57.5, and 60) (Table S2).

The proposed (Method A) achieved a score value which is higher than that of the proposed (Method B), confirming its strong environmental profile due to minimal solvent use and low energy demands of it and reflecting the higher environmental impact associated with Method B’s continuous solvent flow and higher energy consumption, despite an efficient mobile phase.

Therefore, for pesticide residue analysis, while RP-HPLC remains critical for its precision, the adoption of HP-TLC-based approaches is a valuable strategy for mitigating the environmental footprint. The choice of method, or their combination, effectively balances analytical performance with the principles of green analytical chemistry, as demonstrated by the AGREE assessment.

## Conclusion

Selective, precise, and accurate HP-TLC and RP-HPLC methods were developed and validated for the simultaneous determination of chlorfenapyr (CLF), abamectin (ABM), and fenpyroximate (FNP). The HP-TLC method offers a key advantage: simultaneous analysis of multiple samples with minimal mobile phase consumption, enabling rapid and selective pesticide quantification. HP-TLC is cost-effective for pesticide screening (low solvent use, high throughput), while RP-HPLC offers higher sensitivity and regulatory compliance at a greater cost. Labs can combine both for optimal efficiency.

These chromatographic methods are suitable for local and cross-border inspection systems to monitor eggplant samples, ensuring compliance with pesticide residue limits for domestic use or export. The developed simultaneous methods provide a practical solution for regulatory laboratories tasked with monitoring this specific pesticide combination. Compared to running three separate single-analyte methods, our approach reduces analysis time, solvent consumption, labor, and overall cost per sample by approximately two-thirds. This enhances surveillance capabilities by enabling higher throughput screening of samples, ensuring more comprehensive compliance checking with MRLs for this common pesticide combination in a key crop like eggplant. Validation with commercial formulations confirmed that excipients do not interfere with pesticide quantification, demonstrating their utility for quality control of both bulk powders and formulated products.

**Recommended Workflow for Regulatory Application**.

Accordingly, we outline a clear, step-by-step protocol:

1. Sample Preparation: Follow the extraction method detailed in Sect. 2.4.5.
2. High-Throughput Screening: Analyze all samples via the developed HP-TLC method (Method A).
3. Confirmation of Positive Hits: Any sample showing a peak near the MRL must be re-analyzed and confirmed using the more precise RP-HPLC method (Method B).
4. Reporting: Quantify using the HPLC method and report values.

**Table 1:** Validation results and system suitability testing parameters of the methods A and B.

Validation criteria	Values measured						Approved criteria that follow guidelines of the ICH
	Method A			Method B			
	CLF	ABM	FNP	CLF	ABM	FNP	
Linearity, R=	0.99995	0.99997	0.99991	0.99997	0.99993	0.99992	*r* ≥ 099
Slope	2282.90	7439.49	5839.80	420.98	868.59	626.93	
Intercept	0.17	2.59	2.41	−1.79	1.33	−0.68	
Range	0.005–0.50.005.50	0.002–0.10.002.10	0.01–0.12	0.02–2.00.02.00	0.0091.00	0.09–1.20	---
	(µg/band)			(µg/mL)			
Precision (6 replicates)	0.34	0.62	0.59	0.21	0.84	0.71	RSD ≤ 2%
Accuracy (Recovery means ± SD)	100.24% ± 1.043	100.52% ± 1.014	100.19% ± 0.754	100.17% ± 0.702	99.77% ± 1.159	99.99% ± 0.998	100 ± 2%
Specificity R_s_	1.62	5.08		2.08	2.08		complete resolved peaks Resolution is < 1.5
ME %	−8.5			+ 6.2			not significantly interfere with quantification
LOD	0.0015	0.0006	0.0027	0.0063	0.0029	0.0296	Using the formula: 3.3 × SD/slope
	(µg/band)			(µg/mL)			
LOQ	0.0049	0.0019	0.009	0.0191	0.0089	0.089	Using the formula: 10 × SD/slope
	(µg/band)			(µg/mL)			
Robustness	0.79	0.64	0.91	0.48	0.97	0.88	Every alteration should have a pooled RSD of less than 3%

System Suitability Testing Parameters	Method A			Method B			Reference value
	FNP	ABM	CLF	FNP	ABM	CLF	
Tailing factor* (T)	1.31	1.22	1.09	1.17	1.18	1.08	>1.5
Capacity factor (K^’^)	7.41	5.14	2.48	6.98	3.87	2.13	1–10
Resolution (R_S_)	2.71	3.01	5.08	1.62			< 1.5
Selectivity (α)	1.44	2.08	1.80	1.82			< 1
Column efficiency (N)	2254	1254	870	-	-		↑with efficiency of the separation
HETP (cm plate^− 1^)	0.021	0.015	0.087	-	-		↓ the value↑ the column efficiency

HETP = height equivalent to theoretical plate, (cm.plate^− 1^).

^*^Calculated using three peaks.

**Table 2:** Determination of CLF, ABM and FNP in their commercial formulations Baylora^®^, Aquachem^®^ Powder, Ortus super^®^, respectively, by the proposed methods and application of the standard addition technique.

Method A					Method B				
Taken (µg/band)	Found* %	Pure added	Pure Found (µg/band)	R %	Taken (µg mL−)	Found* %	Pure added	Pure Found (µg/band)	R %
CLF									
0.04	99.87	0.03	0.03	100.01	0.4	100.08	0.3	0.29	99.98
		0.05	0.04	99.98			0.4	0.38	98.01
		0.07	0.07	100.12			0.5	0.5	100.54
			Mean ± SD	100.05 ± 0.99				Mean ± SD	99.58 ± 1.21
ABM									
0.02	99.87	0.03	0.02	99.78	0.1	99.69	0.3	0.3	100.12
		0.05	0.05	100.21			0.4	0.4	100.01
		0.07	0.07	99.81			0.5	0.48	98.24
			Mean ± SD	99.54 ± 0.71				Mean ± SD	99.23±0.95
FNP									
0.06	100.34	0.03	0.03	99.62	0.4	100.25	0.3	0.31	100.99
		0.05	0.05	99.81			0.4	0.39	99.67
		0.07	0.06	98.69			0.5	0.51	100.89
			Mean ± SD	99.10 ± 0.51				Mean ± SD	100.02 ± 0.89

*Average of three determinations.

**Table 3:** Statistical comparison of the results obtained by the proposed methods and the reported methods for determination of pure CLF, ABM, and FNP.

Parameter		Method A			Method B			Reported Methods ^(7, 29 & 36)^		
		CLF	ABM	FNP	CLF	ABM	FNP	CLF	ABM	FNP
Mean	100.24	100.52	100.19	100.17	99.77	99.99	99.38	99.20	99.41	
SD	1.04	1.01	0.75	0.70	1.16	0.99	1.31	1.18	1.30	
Variance	1.08	1.02	0.56	0.49	0.45	0.98	1.72	1.39	1.69	
N	7	7	7	7	7	7	7	7	7	
Student’s *t*-test* (2.45)	0.57	0.87	0.45	0.12	0.89	0.09	----	----	----	
*F*- test* (4.28)	1.98	1.68	2.14	1.89	2.01	1.68	----	----	----	

*Figures in parenthesis are the corresponding tabulated values at *p* = 0.05.

## Supplementary Information

Below is the link to the electronic supplementary material.

Supplementary Material 1

## Data Availability

All data generated or analysed during this study are included in this published article.
