# Supplementary material for: Dual chromatographic analysis of pesticide residues in eggplants: a green approach to food safety monitoring
Source: Sci Rep. 2025 Nov 27;15:42546. doi: 10.1038/s41598-025-27091-w (PMC12663312; doi:10.1038/s41598-025-27091-w)
Supplement: Supplementary file 1 — Supplementary Material 1 [file 41598_2025_27091_MOESM1_ESM.docx]

**Supplementary Figures**


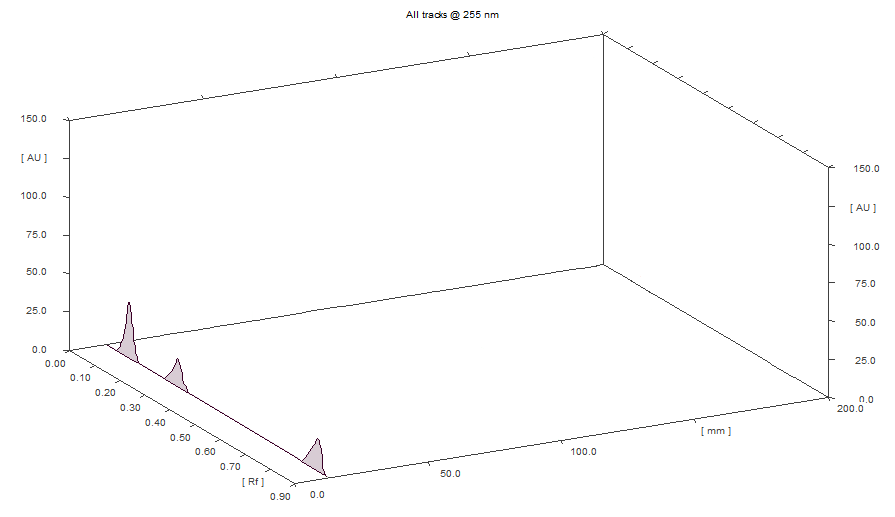
**A**

**B**


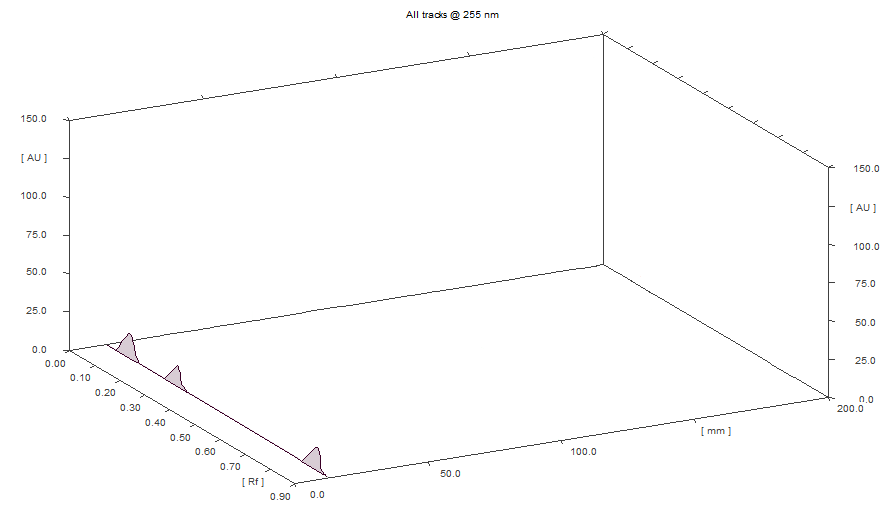


**Figure S1.** 3D HP-TLC-densitogram of CLF (R_f_=0.13), ABM (R_f_=0.34) and FNP (R_f_=0.85) using MeOH:CFM:ACT:GAA:TEA (7.00: 2.50: 0.50: 0.1: 0.1, v/v) as a developing system at 255 nm.

A: In commercial formulation (CLF, ABM, & FNP: 0.009, 0.004 and 0.02 µg/band)

B: in field sample (CLF, ABM, & FNP: 0.008, 0.003 and 0.01 µg/band)


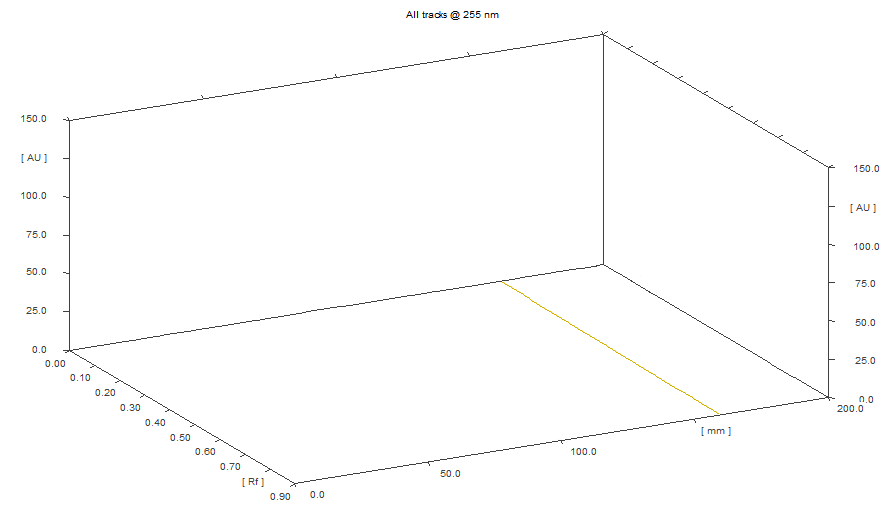


**Figure S2.** 3D HP-TLC-densitogram of blank sample using MeOH:CFM:ACT:GAA:TEA (7.00: 2.50: 0.50: 0.1: 0.1, v/v) as a developing system at 255 nm.

**A B**


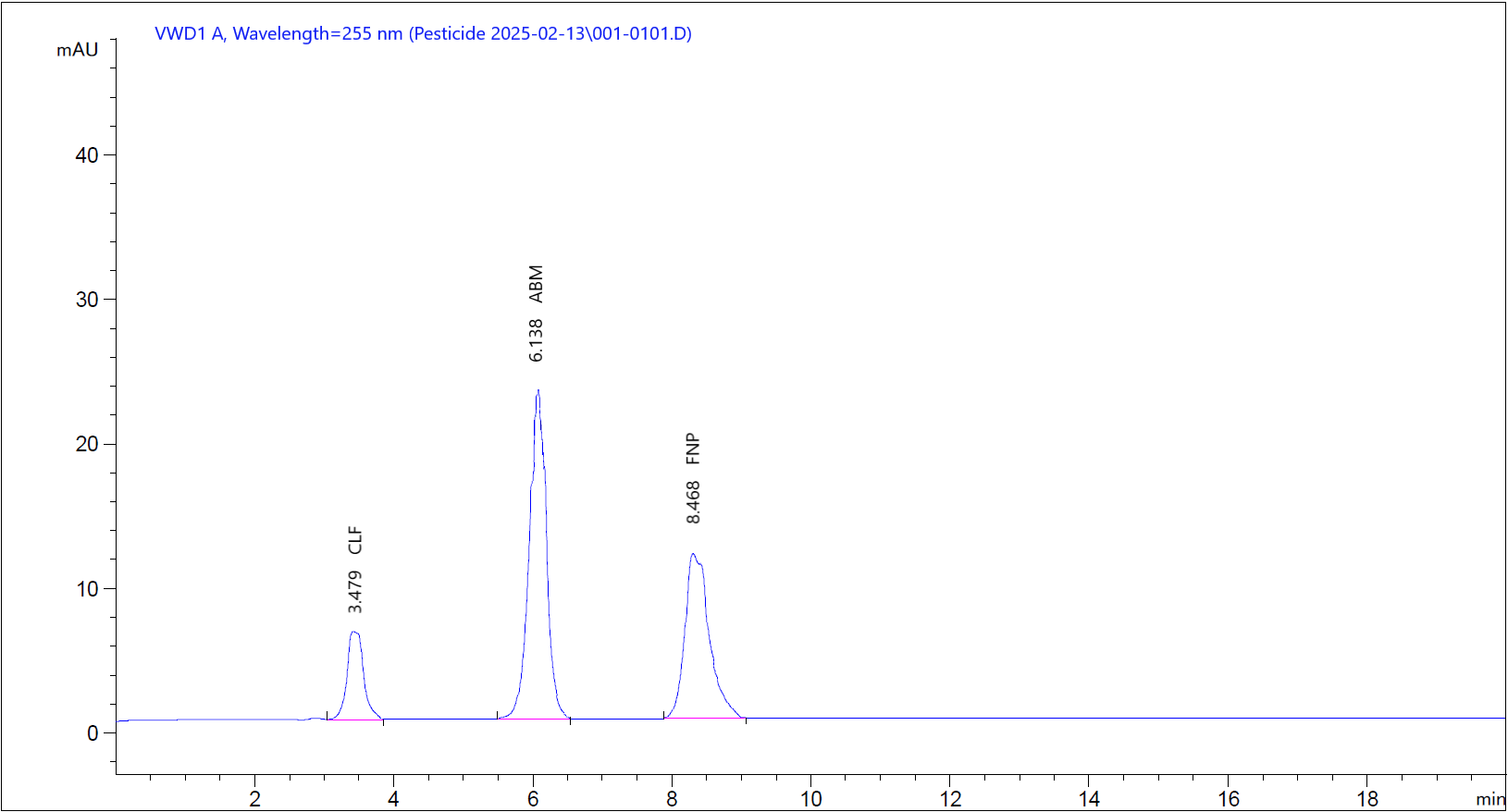

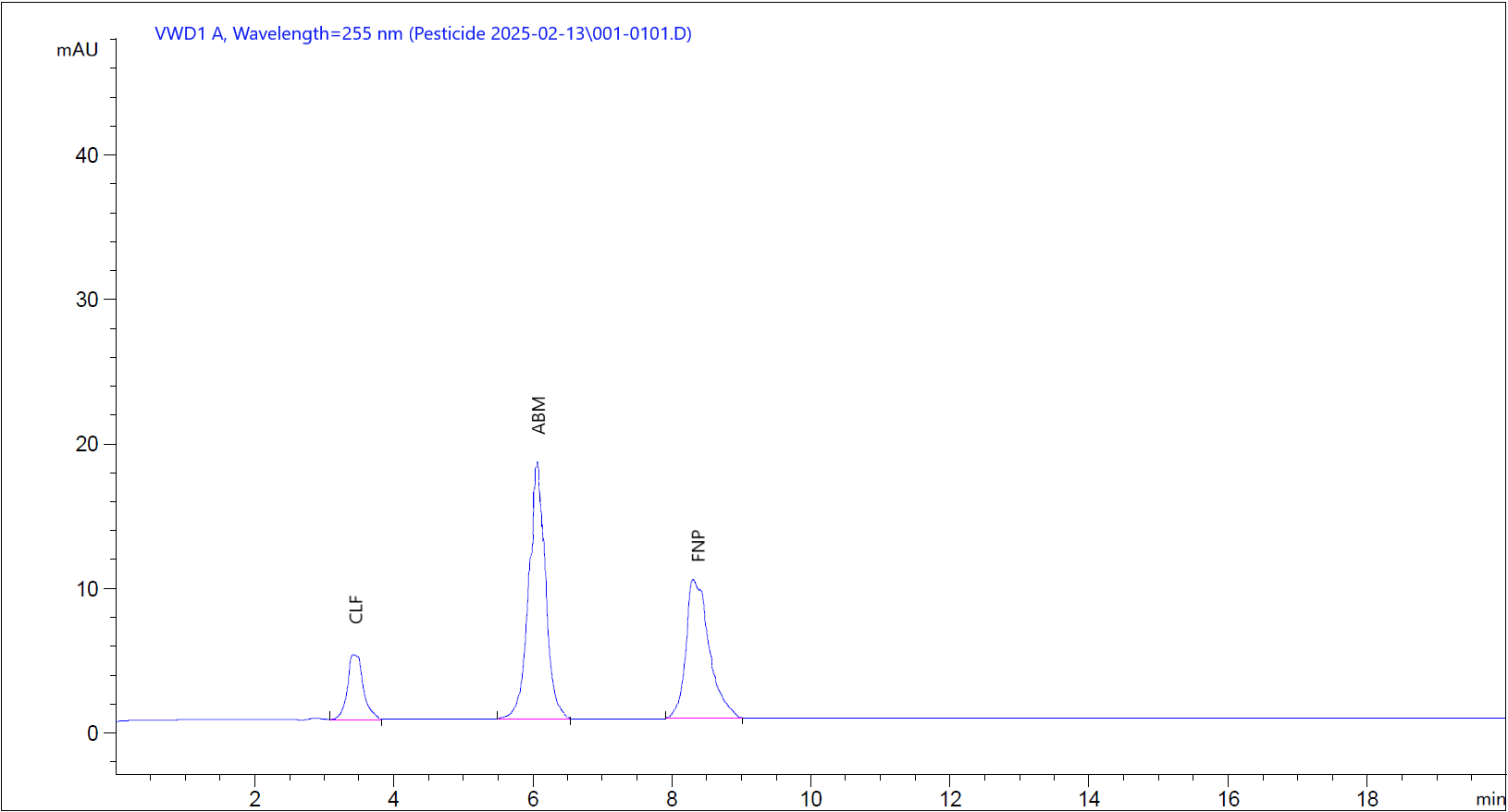


**Figure S3.** . RP-HPLC chromatogram of CLF,ABM, and FNP using ACN: 5 mM AA buffer (70: 30, v/v), pH adjusted to 4.00, flow rate 1.20 mL.min-1 at 255 nm..

A: In commercial formulation (CLF, ABM, & FNP: 0.90, 0.02 and 0.25 µg/mL)

B: in field sample (CLF, ABM, & FNP: 0.80, 0.03 and 0.10 µg/mL)


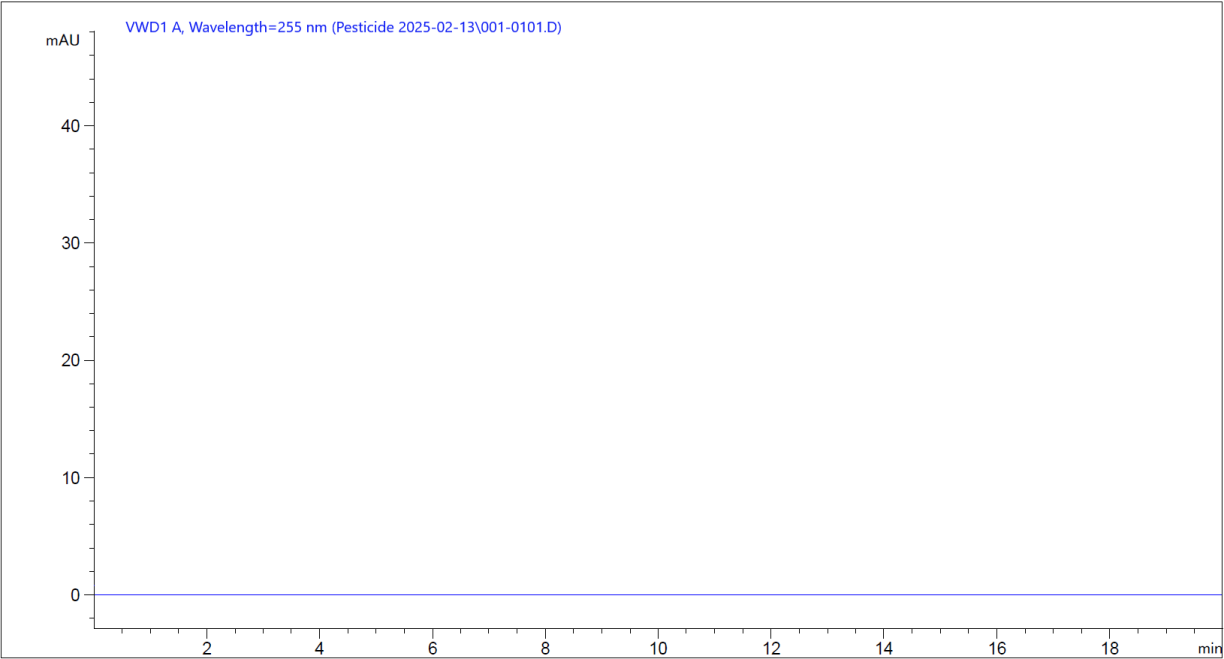


**Figure S4.** HPLC chromatogram of blank sample using ACN: 5 mM AA buffer (70: 30, v/v), pH adjusted to 4.00, flow rate 1.20 mL.min^-1^ at 255 nm.

CLF ABM

FNP

**Figure S5.** claibration graph, Method A.

CLF ABM

FNP

**Figure S6.** claibration graphs, Method B.

**Supplementary Table**

**Table S1:** Field sample analysis for determination of CLF, ABM, and FNP by the proposed methods.

| Pesticides residue at | Residue found* (mg Kg^-1^) | | | | | | | |
| --- | --- | --- | --- | --- | --- | --- | --- | --- |
|  | **Method A** | CLF | ABM | FNP | **Method B** | CLF | ABM | FNP |
| First day |  | 2.091 | 0.067 | 0.089 |  | 1.978 | 0.061 | 0.078 |
| Day 10 |  | 0.821 | 0.003 | 0.108 |  | 0.765 | 0.003 | 0.001 |
| QC** Samples  (Recovery means ±SD) |  | 105.02%  ±1.953 | 104.57%  ±2.234 | 103.39%  ±1.854 |  | 95.25%  ±2.021 | 98.43%  ±2.034 | 96.99%  ±2.001 |
| QC** Samples  RSD |  | 0.876 | 0.452 | 0.921 |  | 0.937 | 0.983 | 0.763 |

*Average of six determinations.

QC** quality control samples for HP-TLC (CLF, AMB, and FNP in concentrations 0.005 , 0.002, and 0.01

(µg/band, respectively) and HPLC (CLF, AMB, and FNP in concentrations 0.02, 0.009, and 0.09

(µg/mL, correspondingly).

**Table S2:** Analytical Greenness (AGREE), Blue Applicability Grade Index (BAGI), and Red Analytical Performance Index (RAPI) comparison of the proposed methods and the reported HPLC methods.

|  | **AGREE** | **BAGI** | **RAPI** |
| --- | --- | --- | --- |
| The proposed RP-HPLC method | 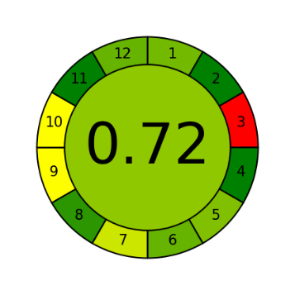 | 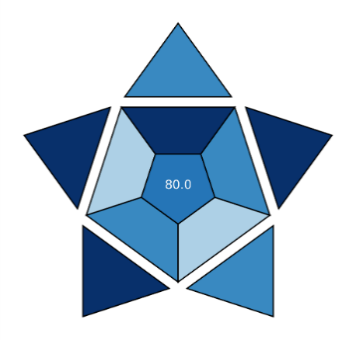 | 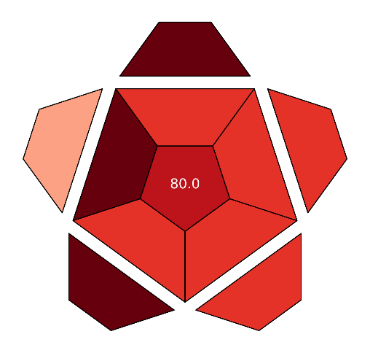 |
| The proposed HP-TLC method | 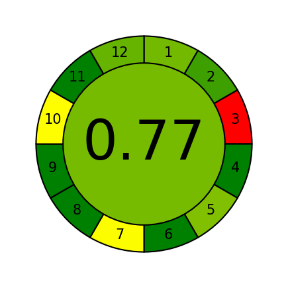 | 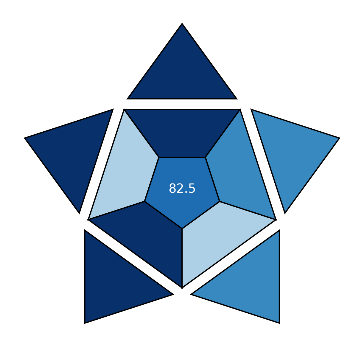 | 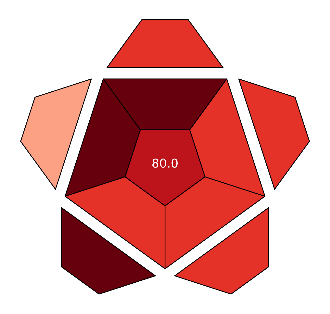 |
| HPLC reported method [Yongsong Cao, Jiuxin Chen, Yuelong Wang, Ji Liang, Lihua Chen, Yitong Lu (2005)], [7] | 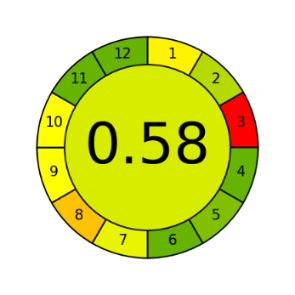 | 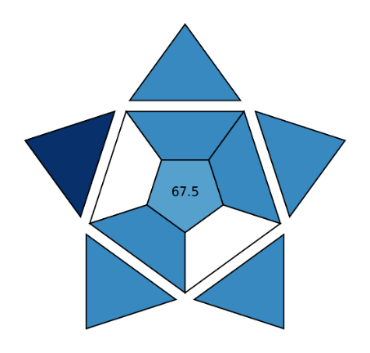 | 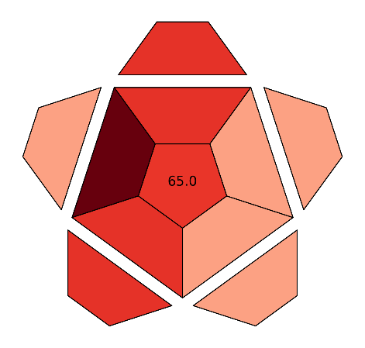 |
| HPLC reported method [Xie, X., Wang, X. & Zhao, L. A (2011)], [29] | 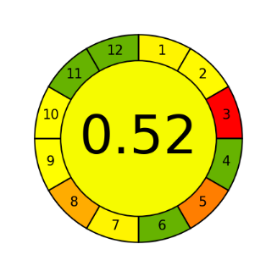 | 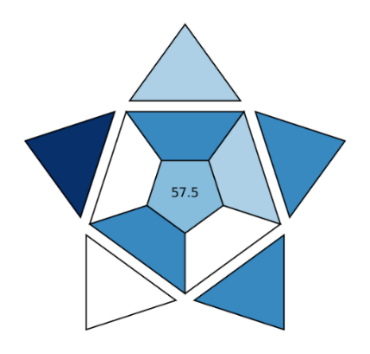 | 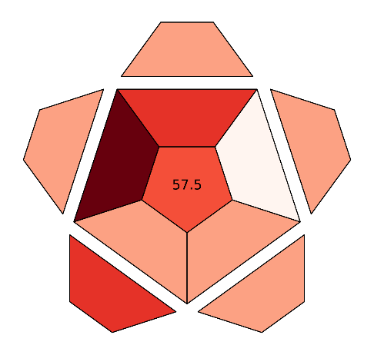 |
| HPLC reported method [Abd Al-Rahman, S.H., Almaz, M.M. & Osama, I.A. (2012)], [36] | 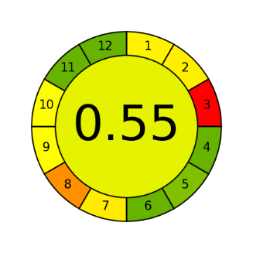 | 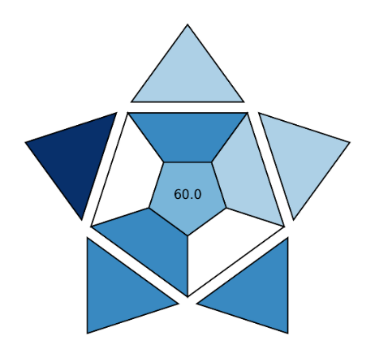 | 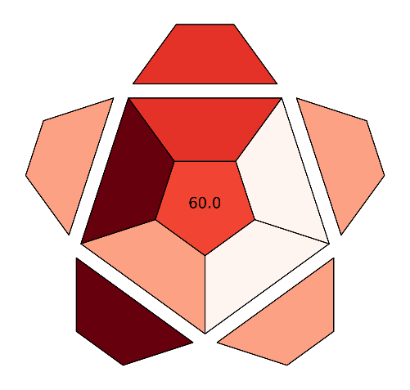 |

**Supplementary Data**

**Cost analysis**

A cost analysis reveals that the consumable cost per sample for HP-TLC screening is approximately $0.85, compared to $4.20 for RP-HPLC analysis, primarily due to RP-HPLC's higher solvent and column costs. This makes HP-TLC a highly cost-effective first-line screening tool. For a lab processing 100 samples weekly, using HP-TLC for initial screening could reduce solvent consumption by ~12 L/week and annual costs by over $15,000, justifying the proposed workflow for labs seeking to balance analytical rigor with economic efficiency.
